# Contribution of Iran to the Ophthalmic Literature over the Past Three Decades

**Published:** 2011-07

**Authors:** Marzieh Katibeh, Hamid-Reza Moein, Mohammad-Ali Javadi

**Affiliations:** 1 Ophthalmic Research Center, Shahid Beheshti University of Medical Sciences, Tehran, Iran; 2 Young Researchers Club, Islamic Azad University, Tehran Medical Branch, Tehran, Iran

**Dear Editor,**

The number of publications plays a major role in assessing scientific progress in different countries. To determine the status of our country in terms of the number of published papers in indexed peer-reviewed medical journals, we searched MEDLINE/PubMed. This database belongs to the National Library of Medicine, the world’s largest biomedical library located in Bethesda, Maryland, USA, which is freely accessible and contains over 18 million journal citations since 1948.^1^ This approach has been applied to ascertain the contribution of different countries to scientific articles in different medical fields as well as ophthalmology.^2^–^5^ We searched PubMed for articles from different ophthalmic institutions which had mentioned their country of origin in the affiliation field. To have a better view, we compared Iran to some other developing, as well as developed countries, in terms of the number of ophthalmic publications. As all countries were not included in this search, the results do not reflect a comprehensive, but rather comparative ranking of different countries. We selected countries presumed to be influential in the field of ophthalmology and vision research. The search results were limited to a 30-year period, from 1981 to the end of 2010.

The total number of indexed papers from ophthalmic institutions during the above mentioned period was 119,640. The overall number of Iranian publications rose dramatically over the recent years, especially during the last decade (Fig. 1). There are 22 nations in the Eastern Mediterranean region (EMR); some countries with a larger number of publications in our search are reported in Table 1. During the same period of time, India and Turkey had 2,820 and 2,401 publications, respectively. Table 2 shows the overall number of ophthalmic publications in some developed countries.

Countries such as India, China and Turkey have demonstrated significant growth in the number of publications and reached volumes exceeding certain developed countries. Similarly, the number of Iranian ophthalmologic publications has shown a significant increase over the past 30 years. In addition to the crude number of publications, citations to a paper is of great importance and shows the impact of that paper on the scientific community.^6^ Most cited articles up to 2006 originated from 41 institutions in 10 countries.^7^

Since PubMed mainly covers English language articles, our statistics may not reflect the exact number of ophthalmologic publications for each country. In addition, it is possible that the name of the country was not mentioned in the affiliation field. However, since these biases were similar for all included countries, one may assume our results to be an acceptable estimate of Iran’s situation in the ophthalmic community during the past decades.

Although the number of Iranian ophthalmologic publications in international peer-reviewed journals has risen significantly, our nation still needs to make a greater contribution to science and to better demonstrate its capabilities. In order to gain a better ranking in the world, we need to extend and improve scientific thinking among our medical care workers which requires a great amount of effort from Iranian ophthalmologists and research centers. Main obstacles to the production of indexed papers in most developing countries include language barriers, instability and incorrect research priorities, as well as the large burden of disease prohibiting the allocation of adequate research budget.^8^,^9^ In order to overcome these limitations, it is crucial to enhance our research capacities by improving human resources, promoting infrastructures (institutional development), optimizing data collection and access, and boosting scientific relations with developed countries. In addition, the role of adequate financing by the government for medical research needs to be emphasized.^10^

## Figures and Tables

**Figure 1:**
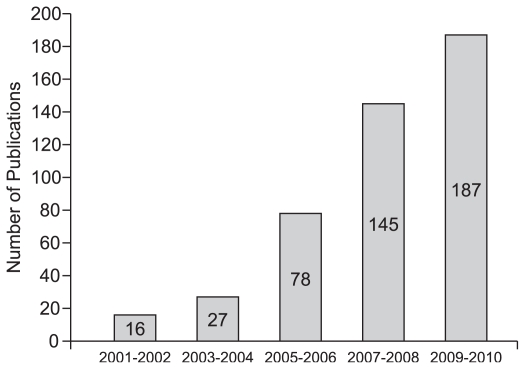
Trend of Iranian ophthalmic publications indexed in PubMed from 2001 to 2010.

**Table 1:** The number of ophthalmic publications indexed in PubMed during the last 30 years in the top 6 countries in the Eastern Mediterranean region

	Country	1981–1990	1991–2000	2001–2010	Total
1	Saudi Arabia	48	296	379	723
2	Iran	0	21	453	474
3	Egypt	14	26	148	188
4	Oman	0	7	133	140
5	Lebanon	7	41	79	127
6	Pakistan	0	4	57	61

**Table 2:** The number of ophthalmic publications indexed in PubMed from 1981 to 2010 in some developed countries

	Country	1981–1990	1991–2000	2001–2010	Total
1	USA	52	11439	22545	34036
2	Japan	732	4874	5947	11553
3	UK	359	2545	5126	8030
4	China	76	812	4569	5457
5	Germany	95	1452	2899	4351
6	Canada	159	829	1214	2202
7	Sweden	174	714	704	1592
8	Switzerland	60	404	785	1249
9	Spain	31	280	556	867
10	France	35	249	544	828
